# Supplementary material for: Building Individual Player Performance Profiles According to Pre-Game Expectations and Goal Difference in Soccer
Source: Sensors (Basel). 2024 Mar 6;24(5):1700. doi: 10.3390/s24051700 (PMC10935308; doi:10.3390/s24051700)
Supplement: Supplementary file 1 [file sensors-24-01700-s001.zip › sensors-2818922-SI.pdf]

Article

Building Individual Player Performance Profiles According to Pre-Game Expectations and Goal Differences in Soccer

Arian Skoki [1], Boris Gašparović [1], Stefan Ivić [2], Jonatan Lerga [1,3,*] and Ivan Štajduhar [1,3]

[1] Department of Computer Engineering, Faculty of Engineering, University of Rijeka, Vukovarska 58, 51000 Rijeka, Croatia; arian.skoki@riteh.uniri.hr (A.S.); boris.gasparovic@riteh.uniri.hr (B.G.); ivan.stajduhar@riteh.uniri.hr (I.Š.)

[2] Department of Fluid Mechanics, Faculty of Engineering, University of Rijeka, Vukovarska 58, 51000 Rijeka, Croatia; stefan.ivic@riteh.uniri.hr

[3] Center for Artificial Intelligence and Cybersecurity, University of Rijeka, R. Matejcic 2, 51000 Rijeka, Croatia

* Correspondence: jonatan.lerga@riteh.uniri.hr

S1. Comprehensive Results Analysis

To highlight the enhancement of the model compared to the baselines B_1 and B_2, Table S1 displays the absolute error of the superior model using NM. The data demonstrate that the model surpasses both baselines for all players, with an average improvement of 6.9% over B_1 and 4.79% over B_2.

The stability of the resultant vectors generated by both PSO and NM was assessed by running each optimization procedure ten times. Table S2 displays the mean and standard deviation values of the final cost function. Notably, both algorithms consistently converge to the final solution across all iterations, as indicated by the minimal standard deviation. Although there are slight variations in the number of evaluations required to reach the optimal solution, these deviations fall within the expected range. These findings underscore the robustness of the proposed approach.

In conclusion, Figure S1 offers an overview of all players and matches included in the study, comparing calculated values to the real ones. This visualization encapsulates the entirety of the analysis conducted.

Table S1. Comparison of NM model vs. baselines B_1 (mean) and B_2 (median), displaying the absolute error for each player. The proposed model consistently outperforms both baselines across all players, showcasing a mean difference of −7.19% for B_1 and −4.79% for B_2. Values in bold indicate the best-performing approaches with the lowest errors.

Player	AE_{NM}	AE_{B_1}	AE_{B_2}	Model vs. B_1 ϵ	Model vs. B_2 ϵ
athlete1	**181848.4**	196257.0	194186.0	**-7.34%**	**-6.35%**
athlete2	**201158.89**	207123.0	204857.0	**-2.88%**	**-1.81%**
athlete3	**192716.72**	212172.0	209929.0	**-9.17%**	**-8.2%**
athlete4	**154328.89**	164862.0	163497.0	**-6.39%**	**-5.61%**
athlete5	**158710.3**	163590.0	158899.0	**-2.98%**	**-0.12%**
athlete6	**215514.32**	231386.0	221867.0	**-6.86%**	**-2.86%**
athlete7	**204385.48**	210075.0	206633.0	**-2.71%**	**-1.09%**
athlete8	**146735.51**	154482.0	151935.0	**-5.01%**	**-3.42%**
athlete9	**114652.47**	124830.0	121886.0	**-8.15%**	**-5.93%**
athlete10	**62925.13**	66654.0	66047.0	**-5.59%**	**-4.73%**
athlete11	**78828.56**	100069.0	109100.0	**-21.23%**	**-27.75%**
athlete12	**33806.67**	36950.0	35137.0	**-8.51%**	**-3.79%**
athlete13	**183325.78**	189136.0	187627.0	**-3.07%**	**-2.29%**
athlete14	**145499.14**	148588.0	143636.0	**-2.08%**	**–1.3%**
athlete15	**134646.25**	144411.0	141562.0	**-6.76%**	**-4.89%**
athlete16	**37304.38**	40011.0	38476.0	**-6.76%**	**-3.05%**
athlete17	**38677.18**	44818.0	43868.0	**-13.7%**	**-11.83%**
athlete18	**35201.96**	36948.0	36458.0	**-4.73%**	**-3.45%**
athlete19	**26778.8**	28854.0	25547.0	**-7.19%**	**–4.82%**
				μ=-6.9%	μ=-4.79%

Table S2. Convergence stability of both PSO and NM methods over ten runs for all players. The data presented include the mean and standard deviations of the final cost function results across the runs (run, ϵ), along with the number of evaluations conducted throughout these runs $(run, eval)$.

Player	$PSO_{run,\epsilon}$	$NM_{run,\epsilon}$	$PSO_{run,eval}$	$NM_{run,eval}$
	μ σ	μ σ	μ σ	μ σ
athlete1	51294.02 214.51	52555.57 2809.51	4859 1597	2660 1870
athlete2	54503.55 54.7	55470.82 3207.25	6139 2414	3317 3739
athlete3	48086.41 1483.52	48372.94 2543.17	4620 1445	1566 443
athlete4	61708.33 24.56	63663.91 2710.29	6425 3591	3627 4580
athlete5	51326.06 207.2	55037.96 8095.42	6334 2814	3574 3527
athlete6	86397.37 119.84	86331.36 13.39	5267 1198	1939 383
athlete7	68422.6 39.5	70558.34 3553.95	5585 2190	2304 902
athlete8	39521.77 2218.21	39458.72 2140.44	6048 4963	2095 508
athlete9	55916.96 77.25	56620.64 1831.9	5071 1933	2139 483
athlete10	52627.32 427.0	54912.22 6457.15	5704 2408	1909 729
athlete11	112301.9 79.7	112256.8 18.56	5331 2092	2467 2108
athlete12	53582.16 3.47	53985.72 1278.91	2456 441	2671 2163
athlete13	42849.12 18.18	46627.51 5172.77	5646 2527	2362 2205
athlete14	71012.21 2711.95	77567.53 21769.11	6008 3627	4160 3992
athlete15	46336.36 30.48	46347.77 66.64	4453 847	2028 395
athlete16	27242.66 10.93	27232.54 0.08	5385 2809	2125 392
athlete17	61759.87 49.33	62548.22 2574.02	4460 2603	1956 721
athlete18	35532.76 11.0	35525.45 0.21	3320 363	1329 629
athlete19	98801.18 7.31	101259.98 6156.62	4084 1709	2001 300

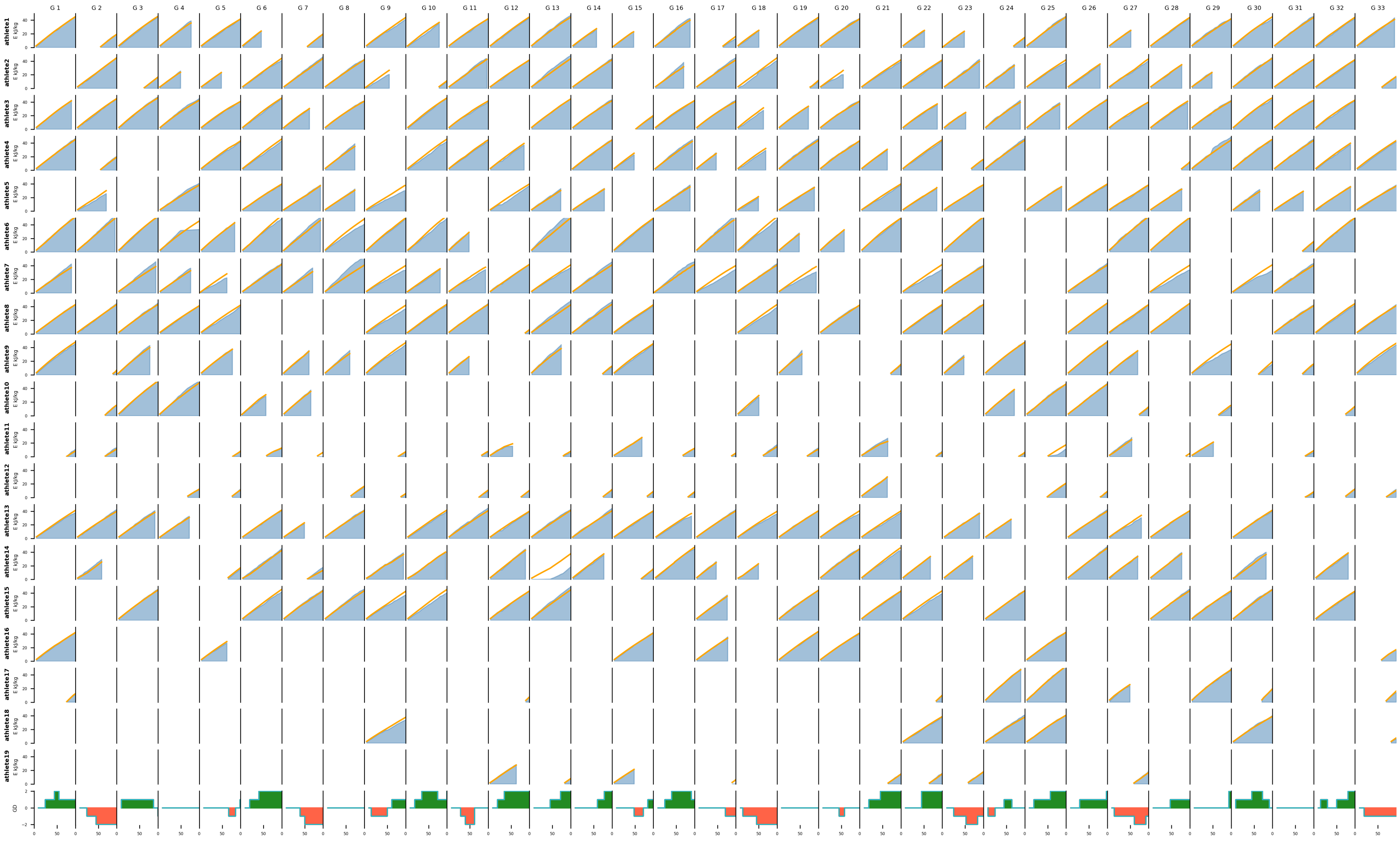

Figure S1. Overview of all the games and players used in the study. Columns represent individual games (G1–G33), while rows correspond to players (athlete1–athlete19), with the last row indicating GD across 90 minutes for each observed game. The negative GD values are highlighted in red, and positive values in green. The orange line reflects calculated values using the proposed model, and the blue area illustrates real values measured via sensors.
